# Reliability and Validity of Alzheimer's Disease Screening With a Semi-automated Smartphone Application Using Verbal Fluency

**DOI:** 10.3389/fneur.2021.684902

**Published:** 2021-07-08

**Authors:** Soon Jai Kwon, Hye Sung Kim, Ji Hyun Han, Jong Bin Bae, Ji Won Han, Ki Woong Kim

**Affiliations:** ^1^Dementia Center, Incheon Sejong Hospital, Incheon, South Korea; ^2^Seongnam Citizens Medical Center, Gyeonggi-do, South Korea; ^3^Yoon's Psychiatry Clinic, Gyeonggi-do, South Korea; ^4^Department of Neuropsychiatry, Seoul National University Bundang Hospital, Gyeonggi-do, South Korea; ^5^Department of Psychiatry, Seoul National University College of Medicine, Seoul, South Korea; ^6^Department of Brain and Cognitive Science, Seoul National University College of Natural Sciences, Seoul, South Korea

**Keywords:** screening, mobile applications, verbal fluency, reliability, validity, Alzheimer's disease

## Abstract

**Introduction:** This study aimed to examine the reliability and validity of Alzheimer's disease (AD) screening with a self-administered categorical verbal fluency test using a semi-automated Android application (app; tCVFT). Furthermore, its diagnostic accuracy concerning AD was compared with both that of a conventional categorical verbal fluency test (cCVFT) administered by a health professional and the Mini-Mental State Examination (MMSE).

**Materials and Methods:** Participants included 100 community-dwelling patients with early AD, whose Clinical Dementia Rating was either 0.5 or 1, and a further 100 sex-matched cognitively normal controls. The internal consistency and test-retest reliability of the tCVFT weighted sum score (tCVFT-WS) was examined using Cronbach's alpha and Pearson's correlation analyses (adjusted for age and education), respectively. The concurrent validity of the tCVFT-WS was examined by testing its correlations with the cCVFT weighted sum score (cCVFT-WS) and MMSE using Pearson's correlation tests. The diagnostic accuracies for early AD of the tCVFT-WS, cCVFT-WS, and MMSE were estimated and compared using receiver operating characteristic (ROC) analyses.

**Results:** The tCVFT-WS exhibited strong internal consistency (Cronbach's alpha = 0.79). However, its test-retest reliability was moderate (*r* = 0.54) owing to the low test-retest reliability of the second-half responses. The patient group exhibited a higher tCVFT-WS than the control group (*p* < 0.001). Correlations between the tCVFT-WS, cCVFT-WS, and MMSE were significant. The tCVFT-WS's area under the ROC curve for AD was 0.861. At its optimal cutoff, the sensitivity and specificity for AD were 0.78 and 0.77, respectively.

**Conclusions:** The self-administered tCVFT-WS, using an Android app, proved valid and reliable at distinguishing people with early AD from cognitively normal controls.

## Introduction

With global population aging, the number of people with dementia (PWD) is also growing rapidly—from 35.6 million in 2010, this number is expected to double every 20 years (1). South Korea is also experiencing a similar situation. According to a nationwide survey on the epidemiology of dementia, the number of PWD is projected to double every 20 years to reach over three million in 2050. Especially, Alzheimer's disease (AD) approximately accounts for two-thirds of overall dementia, making AD the most prevalent form of dementia (2). However, more than half of PWD do not receive a timely diagnosis because in the majority of cases, AD begins insidiously and progresses gradually. Furthermore, accurate and reliable screening tests are not easily available.

Thus, the demand for screening tests that can accurately detect AD in its earlier stages is on the rise. An effective dementia screening test is one that is sensitive and specific enough to identify individuals with cognitive impairment who need further comprehensive evaluation and that can also be quickly and easily administered by a range of health professionals. However, a test that can be reliably self-administered without the assistance of health professionals would be ideal (2). Although the Mini-Mental State Examination (MMSE) is widely employed in dementia screening in both clinical and research settings (3), it has several limitations as a screening instrument: it takes more than 15 min to administer, requires a trained examiner, exhibits limited sensitivity to early-stage dementia, is subject to demographic influences, and cannot be administered to people with motor or visual impairments (4).

Researchers have previously developed a screening test for AD, which made use of a weighted sum score (WS) of the categorical verbal fluency test (CVFT) (5). The CVFT is a popular brief cognitive test that measures semantic memory, generative naming abilities, and executive functions, all of which are frequently impaired in the early stage of dementia; the MMSE is insensitive toward these factors. However, although the CVFT-WS's diagnostic accuracy concerning AD was good, it proved slightly lower than that of the MMSE. Despite this, the CVFT has the advantage of being brief and widely applicable. Consider the following: it can be administered in 2 min, including instructions; it can be administered even to individuals with impaired motor functions; it is easily automated; and it can be administered remotely, without regional disparities regarding phone and Internet coverage.

From the CVFT, we developed an Android application (app) named “Traffic Light for Dementia” (TLiDe; https://play.google.com/store/apps/details?id=com.appmd.dementia.signal). The app guides people through the process of self-administering the CVFT, without any support from health professionals, and provides the test results directly by app, within 1–3 days (5). In the current version of the TLiDe, owing to the voice recognition software's limited accuracy with regard to Korean spoken by older adults, the users' recorded voice responses are not automatically recognized but converted to text by transcribers. The transcribers type the recorded voice responses into the main server, which then automatically calculates the key index scores and WS. Finally, the main server returns the test results to the TLiDe, displaying the probability of AD. Although the entire process were not fully automated since we still need some manpower to convert voice to text data, TLiDe has some strong points that it doesn't need examiner during the test, and by using only voice data we can adjust the screening test to participants stay in remote place.

This study aimed to examine the reliability and validity of CVFT being self-administered using the TLiDe (tCVFT). Furthermore, it compared the tCVFT's diagnostic accuracy for early AD against the accuracy of the conventional CVFT (cCVFT) as well as the MMSE, administered by a neuropsychologist.

## Materials and Methods

### Study Population

The study included 100 AD with a Clinical Dementia Rating (CDR) of 0.5 or 1; they were visitors from the Dementia Clinic of Seoul National University Bundang Hospital. A further 100 sex-matched cognitively normal control participants were included from the Korean Longitudinal Study on Cognitive Aging and Dementia (6). All participants were 60 years or older, community-dwelling, and exhibited adequate visual and hearing abilities, although many wore glasses and some required hearing aids. The exclusion criteria were the presence of any major psychiatric, neurological, or medical disorders that could affect cognitive functions.

### Diagnostic Assessments

Geriatric psychiatrists administered a standardized diagnostic interview pertaining to each participant's detailed medical history, physical and neurological examinations, and laboratory tests; the interview made use of the Korean version of the Consortium to Establish a Registry for Alzheimer's Disease Assessment Packet Clinical Assessment Battery (7), the Mini International Neuropsychiatric Interview (8), and the Cumulative Illness Rating Scale (9). Thereafter, a panel of research psychiatrists diagnosed the relevant participants with AD according to the Diagnostic and Statistical Manual of Mental Disorders–Fourth Edition criteria (10) through consensus diagnostic conferences. Finally, the level of global cognition and severity of symptoms of dementia were evaluated using the Korean version of the MMSE (11) and CDR (12, 13). Participants with CDR composite score 0 was regarded as healthy control, 0.5 was regarded as questionable AD, and 1 was regarded as mild AD.

### Administering the tCVFT and cCVFT

The cCVFT was administered either by research psychologists or trained research nurses, and each participant self-administered the tCVFT using the TLiDe app on a smartphone. Of the total 200 participants, 72 underwent the tCVFT first while the rest opted for the cCVFT first. In order to evaluate the test-retest reliability of the tCVFT, 20 participants (10 cognitively normal controls and 10 PWD) self-administered the tCVFT twice within a 2-h interval.

In both the tCVFT and cCVFT, each participant was asked to generate as many animal names as possible within 60 s. A total score and seven index scores (14) were obtained from each test: (1) the total score included the number of overall correct responses within 60 s, (2) the first-half score included the number of correct responses within the first 30 s, (3) the second-half score included the number of correct responses during the last 30 s, (4) the perseveration score included the number of repetitive responses (correct or incorrect) within 60 s, (5) the intrusion score included the number of non-animal responses within 60 s, (6) the clustering score consisted of the mean cluster size, and (7) the switching score included the number of switches between clusters within 60 s. In cCVFT, all seven indices were calculated manually, and in tCVFT, after the voice data were converted to text data by human transcriber, the rest procedure were proceeded by a program built in application.

A cluster refers to a group of successively generated words belonging to the same subcategory of animal; therefore, the cluster size pertained to the number of correct responses belonging to each subcategory minus 1. Finally, the WS of both the tCVFT and cCVFT was calculated using the previously reported equation: logit (case) = 1.160 + 0.474 × age + 0.003 × age + 0.226 × educational level – 0.089 × first-half score – 0.516 × switching score – 0.303 × clustering score + 0.534 × perseveration score (5).

### Statistical Analysis

Comparisons between the control and patient groups' continuous and categorical variables were made using Student's *t*-tests and chi-square tests, respectively. The internal consistency of the tCVFT was examined with Cronbach's alpha. The test-retest reliability of the tCVFT total score and tCVFT-WS was evaluated using Pearson's correlation analysis, with adjustments for age and education. The criterion validity of the tCVFT was examined by comparing its seven index scores, total score, and WS between the patient and control groups; the comparison was conducted using an analysis of variance (ANOVA) with adjustments for age and education. The effect of the CVFT's administering sequence on the total score and WS of the tCVFT and cCVFT was examined through a repeated-measures ANOVA. Furthermore, the concurrent validity of the tCVFT was examined by evaluating the correlations between the tCVFT-WS, the cCVFT-WS, and the MMSE total score; the evaluation utilized Pearson's correlation analysis, with adjustments for age and education. The diagnostic accuracy of the tCVFT-WS for early AD was examined using receiver operating characteristic (ROC) analysis; its optimal cutoff score for early AD was determined using the Youden index maximum (sensitivity + specificity – 1) (15). The different diagnostic accuracies (regarding early AD) between the tCVFT-WS, cCVFT-WS, and MMSE total score were evaluated by comparing the respective areas under the curve (AUC) of z-tests (16). The ROC analyses were performed with the MedCalc Statistical Software version 19.1 (MedCalc Software, Ostend, Belgium; https://www.medcalc.org; 2019), while all other analyses were performed with SPSS version 18.0 (IBM Corp., Armonk, NY, USA).

### Ethical Considerations

This study was approved by the Seoul National University Bundang Hospital Ethics Committee and Institutional Review Board (B-1706/401-303). The study protocol was in accordance with the relevant guidelines. All individuals provided written informed consent before enrollment, either themselves or via their legal guardians.

## Results

The findings indicate that the AD group was older, less educated, and scored lower on the MMSE than the control group. The AD group showed lower word list immediate memory, word list delayed recall, word list delayed recognition, visual recall, visual recognition, stroop word, stroop color, stroop word-color, and frontal assessment battery scores than the control group. The AD group also took a longer time to complete the Trail Making Test, both A and B, than the control group. Thus, the AD group showed significantly impaired performance in both memory and executive function. The proportion of participants who performed the tCVFT before the cCVFT was higher in the patient group than in the control group (Table 1). In the repeated-measures ANOVA, the tCVFT and cCVFT total scores were significantly influenced by the administering sequence (F = 6.784, *p* = 0.01), while the WS were not (F = 0.01, *p* = 0.753).

**Table 1:** Characteristics of the participants.

	**Control (*n* = 100)**	**AD patients** **(*n* = 100)**	p^*^
Age (years, mean ± SD)	74.4, 4.8	78.9, 4.8	<0.001
Sex (male, %)	25	25	1.00
Education (years, mean ± SD)	12.3, 4.4	9.3, 5.5	<0.001
MMSE (points, mean ± SD)	27.5, 2.5	18.3, 5.2	<0.001
Word list immediate memory	19.3, 4.3	10.5, 3.4	<0.001
Word list delayed recall	6.3, 2.0	0.89, 1.4	<0.001
Word list delayed recognition	9.3, 1.0	4.2, 3.1	<0.001
Visual recall	7.8, 2.5	1.4, 2.0	<0.001
Visual recognition	3.6, 0.7	2.0, 1.2	<0.001
Trail Making Test A (seconds)	47.4, 28.1	129.6, 98.4	<0.001
Trail Making Test B (seconds)	134.5, 70.6	297.5, 91.9	<0.001
Stroop word	79.4, 14.8	56.0, 18.7	<0.001
Stroop color	66.3, 12.1	45.9, 15.4	<0.001
Stroop word-color	42.0, 10.2	23.4, 11.9	<0.001
Frontal assessment battery	15.9, 2.0	11.02, 3.5	<0.001
Administration (tCVFT first, %)	29	43	0.04

*AD, Alzheimer's disease; SD, standard deviation; MMSE, Korean version of the Mini-Mental State Examination; CVFT, categorical verbal fluency test; tCVFT, semi-automatically administered categorical verbal fluency test*.

* *Student's t-test for continuous variables and chi-square test for categorical variables*.

The tCVFT exhibited strong internal consistency (Cronbach's alpha = 0.79), while its test-retest reliability was good for the total score (*r* = 0.80, *p* < 0.001) and only moderate for the WS (*r* = 0.54, *p* = 0.022). When the test-retest reliability of the seven index scores was analyzed separately, the test-retest reliability of the first-half score was good (*r* = 0.87, *p* < 0.001) while that of the second-half score (*r* = 0.26, *p* = 0.293) was very low. When the test-retest reliability was reanalyzed using only the first-half responses, the WS's test-retest reliability improved (*r* = 0.84, *p* < 0.001).

As summarized in Table 2, the tCVFT-WS was significantly negatively correlated with the tCVFT total score (*r* = −0.82, *p*<0.001), cCVFT total score (*r* = −0.67, *p* < 0.001), and MMSE score (*r* = −0.50, *p* <0.001) and positively correlated with the cCVFT-WS (*r* = 0.61, *p* < 0.001).

**Table 2:** Correlations between the semi-automatically administered categorical verbal fluency test, conventionally administered categorical verbal fluency test, and Mini-Mental State Examination.

**Tests**	**tCVFT-WS**	**tCVFT-T**	**cCVFT-WS**	**cCVFT-T**	**MMSE**
tCVFT-WS	1				
tCVFT-T	−0.82^*^	1			
cCVFT-WS	0.61^*^	−0.63^*^	1		
cCVFT-T	−0.67^*^	0.70^*^	−0.77^*^	1	
MMSE	−0.50^*^	0.57^*^	−0.57^*^	0.62^*^	1

*tCVFT-WS, semi-automatically administered categorical verbal fluency test weighted sum score; tCVFT-T, semi-automatically administered categorical verbal fluency test total score; cCVFT-WS, conventionally administered categorical verbal fluency test weighted sum score; cCVFT-T, conventionally administered categorical verbal fluency test total score; MMSE, Mini-Mental State Examination*.

* *p < 0.001 by Pearson's correlation analyses adjusted for age and education*.

The AD group exhibited a higher tCVFT-WS than the control group. All subindex scores, except the perseveration score, of the AD group were significantly different from the control group's scores (Table 3).

**Table 3:** Performances on the semi-automatically administered categorical verbal fluency test.

	AD patients (*n* = 100)^*^	**Control (*n* = 100)**^**†**^	**p**^**‡**^
Weighted sum score	2.3 ± 1.4	−0.1 ± 1.7	<0.001
Total score	7.7 ± 3.6	16.7 ± 4.6	<0.001
First-half score	6.1 ± 2.5	12.3 ± 3.1	<0.001
Second-half score	1.8 ± 1.9	4.3 ± 2.5	<0.001
Clustering score	2.0 ± 1.7	1.7 ± 1.3	<0.001
Switching score	2.9 ± 1.9	7.7 ± 3.6	<0.001
Perseveration score	1.2 ± 1.4	1.0 ± 1.2	0.55
Intrusion score	0.3 ± 0.5	1.0 ± 1.3	<0.001

* *Early AD with a Clinical Dementia Rating of 0.5 or 1*.

† *Sex-matched cognitively normal controls with a Clinical Dementia Rating of 0*.

‡ *Analyses of variance adjusted for age and education*.

**Figure 1:**
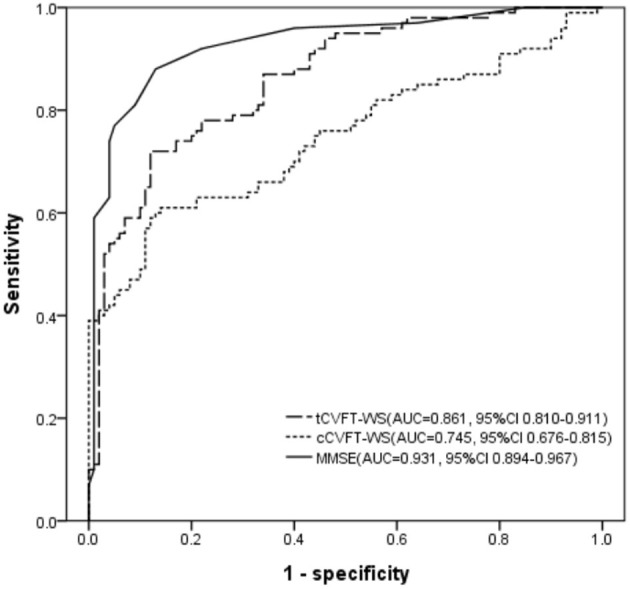
Receiver operating characteristic curve, regarding early AD, of the semi-automatically administered categorical verbal fluency test, conventionally administered categorical verbal fluency test, and Mini-Mental State Examination. tCVFT-WS, semi-automatically administered categorical verbal fluency test weighted sum score; cCVFT-WS, conventionally administered categorical verbal fluency test weighted sum score; MMSE, Mini-Mental State Examination; AUC, area under the receiver operating characteristic curve; CI, confidence interval.

The AUC of the tCVFT-WS was 0.861, indicating that its diagnostic accuracy for AD was good (Figure 1). Although the tCVFT's AUC for AD was smaller than that of the MMSE (0.861 vs. 0.931, *p* = 0.04), it was larger than that of the cCVFT-WS (0.861 vs. 0.745, *p* < 0.001). At its optimal cutoff score, the tCVFT-WS's sensitivity and specificity for early AD was 0.78 and 0.77, respectively (Table 4).

**Table 4:** Diagnostic accuracies, regarding early Alzheimer's disease, of the semi-automatically administered categorical verbal fluency test, conventionally administered categorical verbal fluency test, and Mini-Mental State Examination.

**Test**	**Cutoff**	**SE**	**SP**	**PPV**	**NPV**	**AUC**
tCVFT-WS						0.861
	1.101	0.780	0.750	0.757	0.773	
	1.115	0.780	0.760	0.765	0.776	
	1.143^*^	0.780	0.770	0.772	0.778	
	1.241	0.780	0.780	0.780	0.780	
	1.325	0.770	0.780	0.778	0.772	
cCVFT-WS						0.745
	2.095	0.660	0.620	0.635	0.646	
	2.111	0.660	0.630	0.641	0.649	
	2.116^*^	0.660	0.640	0.647	0.653	
	2.121	0.660	0.650	0.653	0.657	
	2.132	0.660	0.660	0.660	0.660	
MMSE						0.931
	23.5	0.950	0.770	0.805	0.939	
	24.5	0.910	0.810	0.827	0.900	
	25.5^*^	0.870	0.880	0.879	0.871	
	26.5	0.780	0.920	0.907	0.807	
	27.5	0.600	0.960	0.938	0.706	

*tCVFT-WS, semi-automatically administered categorical verbal fluency test weighted sum score; cCVFT-WS, conventionally administered categorical verbal fluency test weighted sum score; MMSE, Mini- Mental State Examination; SE, sensitivity; SP, specificity; PPV, positive predictive value; NPV, negative predictive value; AUC, area under the receiver operating characteristic curve*.

* *Optimal cutoff score*.

## Discussion

The increasing use of mobile technology has made dementia detection more convenient and cost-effective. In a recent review, strategies pertaining to the things to consider when developing accurate and convenient mobile testing/screening devices were categorized into three groups: (1) mobile versions of existing articles or computerized neuropsychological tests; (2) new cognitive tests developed specifically for mobile platforms; and (3) the use of new types of data for cognitive assessments (17). The TLiDe was developed in accordance with all three strategies—the TLiDE is a mobile version of the CVFT, an existing neuropsychological test (5); the CVFT-WS is an improvement over the CVFT, made using the previously reported equation; and the TLiDE uses only recorded vocal data. Validity and reliability were demonstrated in the current study.

The tCVFT's diagnostic accuracy, sensitivity, and specificity were found to be acceptable with regard to screening for early AD (18). Furthermore, in line with our previous work (5, 19), the early AD group exhibited several qualitative differences from the cognitively normal control group, such as lower switching and intrusion scores but a higher clustering score. These results indicate that the tCVFT-WS may be able to reflect these qualitative differences. Although the tCVFT's diagnostic accuracy for early dementia was lower than that of the MMSE—which is administered by a neuropsychologist—the tCVFT still has several advantages as an early dementia screening tool over the MMSE. First, administering the tCVFT is a far briefer process; it takes only 3 min, including instructions and practice trials, as opposed to the MMSE's 15 min (administered by trained health professionals). Second, the tCVFT can be fully self-administered through the TLiDe app since the instructions and responses are simple and deliverable by voice—without any graphical illustration. However, the complex instructions and response methods associated with implementing screening tests through mobile technologies are often challenging for older adults (20–22). Nevertheless, the MMSE requires more complex instructions and input devices to respond to several items, such as “copying a pair of intersecting pentagons” or “three-stage command,” that are not easily self-administered through a mobile app. Third, the tCVFT can be self-administered over more simplistic platforms, such as regular mobile phones, since the instructions and responses function through a voice-only process. This is a significant advantage as it can be widely implemented regardless of regional information technology infrastructure. Fourth, the tCVFT may be far cheaper than the MMSE, which improves the cost-effectiveness of dementia screening services.

Thabtah et al. reviewed 20 mobile app-based dementia screening tools in 2020 (23). Most reviewed tools were developed based on clinically accepted tests such as the MMSE and Montreal Cognitive Assessment. However, these have some limitations. Some of them have complex visual instructions and responses that are not easily self-administered through a mobile app. Others, especially the Dementia & Alzheimer's Memory Diagnosis Test, the mobile version of the MMSE (https://play.google.com/store/apps/details?id=com.alzheimers_mme), covers only a part of the MMSE and is not a comprehensive alternative to the conventional instrument. The TLiDe, which requires simple instruction and uses only voice data, is expected to be an appropriate mobile device-based screening tool.

In the estimation of the WS, the second-half score was not included because of the limited number of retrieved words. In line with the current study, in a voxel-based symptom mapping study (24), the number of retrieved words in the last part of the verbal fluency test was much lower than that in the early part.

The test-retest reliability of the tCVFT-WS was only moderate, which may be attributable to the low test-retest reliability of the second-half score. In the tCVFT, approximately three-fourths of the total correct responses were generated in the first 30 s (79.2% in the AD group and 73.7% in the control group). The test-retest reliability of the first-half score was good (*r* = 0.87, *p* < 0.001) while that of the second-half score was very low (*r* = 0.26, *p* = 0.293). Although the second-half score was excluded from WS calculations, its low test-retest reliability could have reduced the test-retest reliabilities of other index scores that were included in the calculations, such as the switching score, clustering score, and perseveration score. However, when we re-estimated the WS using the responses from the first 30 s, the tCVFT's test-retest reliability improved. Accordingly, future research needs to investigate the diagnostic comparability of the 30- and 60-s tCVFT.

This study has several limitations. First, the proportion of participants who performed the tCVFT before the cCVFT in the patient group was higher than in the control group (43 vs. 29%). However, the administering sequences did not influence the tCVFT-WS and cCVFT-WS. Second, age and education were not matched between the patient and control groups. Although these were controlled in comparing means or variances between groups and testing correlations between variables, they were not controlled in ROC analysis, which might have exaggerated the diagnostic performance of the tests. Third, although we did not evaluate history of smartphone use in the participants, the control group, which was more educated and had better cognition, may have been better at the use of the TLiDe than the patient group. This potential difference in familiarity with smartphones may have contributed to the difference in test performance between the groups. Fourth, since the test-retest reliability of the second-half score was very low, it might have reduced both the test-retest reliability and the diagnostic accuracy of the tCVFT. Therefore, in future research, we need to examine whether a 30-s tCVFT version may improve both accuracy and reliability.

Despite these limitations, considering its several strengths such as brevity, price, and accessibility, we believe the tCVFT-based TLiDe app can be an attractive alternative to the MMSE as a screening instrument or even be used as a pre-screening test before administering the MMSE.

## Conclusions

In conclusion, self-administering the tCVFT through the TLiDe app is a brief, valid, and reliable method for screening for early AD. Furthermore, it may contribute toward improving the cost-effectiveness of dementia screening services and overcoming the regional disparity in dementia screening service access.

## Data Availability Statement

The raw data supporting the conclusions of this article will be made available by the authors, without undue reservation.

## Ethics Statement

The studies involving human participants were reviewed and approved by Seoul National University Bundang Hospital Ethics Committee and Institutional Review Board. The patients/participants provided their written informed consent to participate in this study.

## Author Contributions

SK and KK contributed to drafting the manuscript and the figures. SK, HK, JHH, JB, JH, and KK contributed to the study concept and design and contributed to the analysis and interpretation of data. SK, HK, JHH, and KK contributed to data acquisition. All authors contributed to the article and approved the submitted version.

## Conflict of Interest

The authors declare that the research was conducted in the absence of any commercial or financial relationships that could be construed as a potential conflict of interest.
